# Self-detection of atrial fibrillation in an aged population: three-year follow-up of the LietoAF intervention study

**DOI:** 10.1186/s12877-017-0607-0

**Published:** 2017-09-16

**Authors:** Jussi Jaakkola, Raine Virtanen, Tuija Vasankari, Marika Salminen, K.E. Juhani Airaksinen

**Affiliations:** 1 Heart Centre, Turku University Hospital and University of Turku, Kiinamyllynkatu 4-8, Box 52, FIN-20521 Turku, PO Finland; 2 Institute of Clinical Medicine, Family Medicine, University of Turku, Kiinamyllynkatu 4-8, 20521 Turku, Finland

**Keywords:** Atrial fibrillation, Asymptomatic conditions, Screening, Stroke

## Abstract

**Background:**

Atrial fibrillation (AF) is often asymptomatic and undiagnosed until an ischaemic stroke occurs. An irregular pulse is a key manifestation of AF. We assessed whether pulse self-palpation is feasible in screening of AF.

**Methods:**

Altogether 205 residents of Lieto municipality aged ≥75 years were randomized in 2012 to receive brief education on pulse palpation focusing on evaluating rhythm regularity. Self-detected pulse irregularity and new AF diagnoses were recorded, and the subjects’ quality of life and use of health care services were assessed during a three-year follow-up.

**Results:**

The subjects’ median age was 78.2 [3.8] years, and 89 (43.4%) were men. Overall, 139 (68%) subjects had initial good motivation/capability for regular palpation. At four months, 112 (80.6%) subjects with good and 26 (39.4%) with inadequate motivation/capability palpated their pulse daily. At 12 months, 120 (58.5%) and at 36 months, 69 (33.7%) subjects palpated their pulse at least weekly. During the intervention, 67 (32.7%) subjects reported pulse irregularity. New AF was found in 10 (4.9%) subjects, 7 (70%) of whom had reported pulse irregularity. Pulse irregularity independently predicted new AF, but only one (0.5%) subject with new AF sought undelayed medical attention due to pulse irregularity. Quality of life and number of outpatient clinic visits remained unchanged during follow-up.

**Conclusion:**

Pulse palpation can be learned also by the elderly, but it is challenging to form a continuing habit. The low persistence of pulse self-palpation limits its value in the screening of AF, and strategies to promote persistence and research on alternative screening methods are needed.

**Trial registration:**

http://www.ClinicalTrials.gov identifier: NCT01721005. The trial was registered retrospectively on October 26, 2012.

**Electronic supplementary material:**

The online version of this article (10.1186/s12877-017-0607-0) contains supplementary material, which is available to authorized users.

## Background

Atrial fibrillation (AF) is a major risk factor of thromboembolism and ischaemic stroke [1], and its prevalence in ischaemic stroke patients is 15–25% [2, 3]. Moreover, in a third of all ischaemic strokes no discernible causative factor can be identified despite adequate diagnostic evaluation, and thus these strokes are called cryptogenic [4]. Paroxysmal, asymptomatic AF is probably the underlying aetiology in a significant share of cryptogenic strokes: two recent randomised controlled studies uncovered new unrecognised AF during long-term monitoring in 9–16% of patients with cryptogenic stroke [5, 6]. The prevalence of AF in the general adult population is approximately 1%, but it sharply increases with age, and 9% of those over the age of 80 suffer from the condition [7]. As a consequence of the continuing aging of the population, conditions predisposing to AF are becoming more common, and the prevalence of AF is expected to increase significantly in the future [7, 8], as is the number of thromboembolic complications associated with it [9].

Approximately 10–40% of AF is asymptomatic or ‘silent’ [10] and as a result ischaemic stroke is too often the first clinical manifestation of AF [11]. Oral anticoagulation has proven effective in the prevention of thromboembolism [12]. To facilitate the timely introduction of preventive measures, early detection is paramount, and widely practicable and inexpensive measures to screen for AF are needed. The 2012 focused update of the European Society of Cardiology guidelines on the management of AF recommends opportunistic pulse palpation in patients aged 65 years or older, followed by the recording of ECG for screening of AF [13].

The aim of the LietoAF Study was to assess the feasibility of using pulse self-palpation in screening of AF in an aged population [14]. Early results were encouraging regarding that self-monitoring of heart beat taught by a nurse might be beneficial in the early detection of asymptomatic AF. In this paper, we report the persistence of the habit of regular pulse palpation and the yield of the intervention and its effect on quality of life during a follow-up of three years.

## Methods

### Patient population

**Fig. 1:**
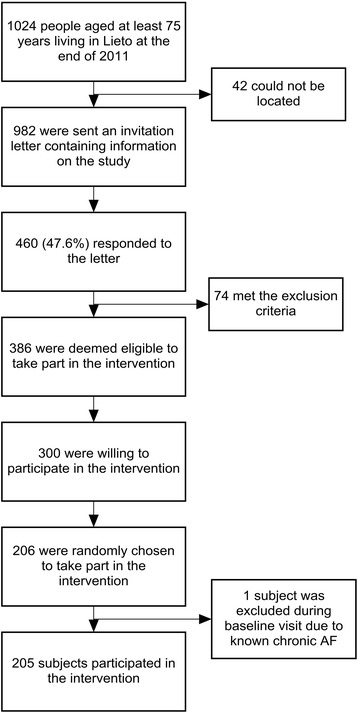
A flow diagram of the target population and subject selection process. Abbreviations. AF: atrial fibrillation

The target population and study sample allocation have been described previously in detail [14]. Briefly, subjects were recruited from among all persons aged at least 75 years living in Lieto, a municipality in South-Western Finland, at the end of 2011 according to information provided by the Finnish Population Register Centre. People with history of chronic AF, who were receiving oral anticoagulants or who needed permanent institutional care were excluded. Finally, there were 205 subjects who were randomly selected to participate in the study from among 300 willing and eligible subjects. A flow diagram depicting the target population and complete subject selection process is presented in Fig. 1.

### Study protocol

The complete study protocol is planned to last up to 5 years and is described in detail in www.ClinicalTrials.gov (Identifier: NCT01721005). The study participants were invited to a health centre for a baseline visit, during which information was recorded on the subjects’ medical history, medication, lifestyle factors, social factors and previous education. Key physical measurements were performed (height, weight, waist circumference and blood pressure) and ECGs recorded. A validated EQ-5D-3L health state questionnaire [15] was completed and Mini-Mental State Examination (MMSE) performed.

A single trained cardiac nurse gave an education on pulse palpation according to a standardised protocol focusing on the evaluation of heart rate and rhythm regularity. First, the subjects received information on the health implications of AF (i.e. risk of thromboembolism and ischaemic stroke), its clinical presentation and frequent asymptomatic nature. The potential health benefits of the intervention were explained (i.e. catching asymptomatic AF and timely implementation of anticoagulation therapy to avoid stroke). Then the nurse taught the technique of pulse palpation with special focus on assessing heart rate and rhythm regularity. In the third phase, the subjects freely palpated their radial and carotid pulses and it was recorded which pulses they could find and which fingers they used for palpation. In the final phase the nurse assessed the achieved capability of pulse palpation. The subjects were deemed to have good capability, if they could find their pulse at a preferred site, count out loud in time with it and assess whether it was regular or irregular, while the study nurse simultaneously palpated the pulse at another site. Capability was deemed inadequate if one or more of these tasks failed. The time spent at each phase varied individually as needed, but the maximum time spent on education was ten minutes.

The participants were asked to continue self-assessment of pulse on a permanent basis twice-daily and when symptoms are noticed. They received a follow-up diary where they should record their heart rate for four months to encourage habit formation. In case of detected pulse irregularity or tachycardia (heart rate at least 110), the participants were told to repeat pulse palpation in 20 min’ time and if the finding should persist to contact healthcare professionals without any periodicity.

After one month, the participants arrived for the first follow-up visit purposed to assess the habit of heart beat self-monitoring and to repeat pulse palpation education, if needed. The subjects’ motivation for regular pulse palpation was assessed based on the completeness of follow-up diary entries. If at least 80% of the possible entries, assuming twice-daily palpation, were recorded, motivation was graded as good, and if less than 80% were completed, motivation was graded as inadequate. Two groups were then formed based on achieved capacity and motivation for regular palpation. If both pulse palpation capability and motivation were graded as good, the subject was classified as having good motivation/capability (*n* = 139), otherwise as having inadequate motivation/capability (*n* = 66). An EQ-5D-3L health state questionnaire was repeated. Possible outcome events, health problems, use of healthcare services and medication were asked, and outcome events were also searched from hospital and community health centre records. The participants were encouraged to continue regular self-assessment of pulse.

At four, 12 and 36 months, mailed questionnaires were used to inquire about pulse palpation activity, detected irregular pulse, healthcare contacts, outcome events and medication. Outcome events were also searched from hospital and community health centre records. A validated EQ-5D-3L health state questionnaire, which records five different dimensions of quality of life (mobility, self-care, usual activities, anxiety/depression and pain/discomfort) as well as the self-reported overall state of health, was repeated at each follow-up to assess the effect of the intervention on quality of life. The EQ-5D-3L health states summary index was calculated to reflect the participants’ overall health applying the Danish time trade-off (TTO) valuation technique to ensure the Scandinavian societal perspective.

Altogether 20 subjects with detected pulse irregularity were selected in an additional intervention where they made a 10 min’ one-lead ECG recording twice a day and when pulse irregularity was detected using a portable ECG recorder which records a high-quality ECG (resolution 2 microvolts, bandwidth 0.05–250 Hz) (Beat2Phone; manufacturer: VitalSignum Oy; http://www.beat2phone.com/en). For recording, the subjects wore a belt, which contained the ECG electrodes and a transmitter, around their chest. The recorded ECGs were transferred via Bluetooth to a smart phone app and a cloud service to be analysed by the investigators. ECG interpretation was visual, and an experienced cardiologist (KEJA) was responsible for all interpretations. Although Beat2Phone has not been formally validated against a 12-lead ECG in diagnosing AF, the ECG-signal was clear, and no difficulties in interpretation were encountered in any of the recordings made by the subjects during this study.

### Outcomes

The primary outcomes of this study were self-detected pulse irregularity and diagnosis of new AF. AF was diagnosed by a 12-lead ECG according to the standard criteria. The secondary outcomes were occurrence of ischaemic stroke or transient ischaemic attack (TIA), implantation of pacemaker due to bradyarrhythmia and death during the follow-up. Ischaemic stroke was defined as a permanent focal neurological deficit adjudicated by a neurologist and confirmed by computed tomography or magnetic resonance imaging. TIA was defined as a transient (<24 h) focal neurological deficit adjudicated by a neurologist. An independent cardiologist assessed the indication for pacemaker. Because of the relatively small sample size, a composite outcome variable consisting of new AF, TIA/ischaemic stroke, death and bradyarrhythmia requiring pacemaker implantation, was used to study whether capability of pulse self-monitoring and self-detected pulse irregularities might have prognostic implications.

### Study ethics

The study protocol was approved by the Medical Ethics Committee of The Hospital District of Southwest Finland. All patients enrolled in the study provided written informed consent for participation. The study conforms to the Declaration of Helsinki.

### Statistical analyses

The statistical analyses were performed with IBM SPSS Statistics software (version 22.0, SPSS, Inc., Chicago, Illinois). Continuous data are presented as median [interquartile range] or mean ± standard deviation and categorical variables as absolute number and percentage. Chi-square test and Fisher’s exact test were used to compare differences between proportions. Mann-Whitney U test and independent-samples t-test were used for the analysis of continuous variables. For repeated measures, one-way ANOVA with repeated measures and Friedman test were used to analyse continuous variables and Cochran’s Q test to analyse dichotomous variables. After the Friedman tests, Wilcoxon Signed Rank test was used in the post-hoc analyses, and Bonferroni correction was applied to the post-hoc two-sided levels of significance. Based on the results of bivariable comparisons, logistic regression analyses were conducted to analyse the independent predictors of the outcome events and continuing pulse palpation activity. Differences were considered significant if the null hypothesis could be rejected at the 0.05 probability level. Analyses were conducted according to the intention-to-treat principle.

## Results

The study population consisted of 205 participants, whose median age was 78.2 [3.8] years, and of whom 89 (43.4%) were men. Altogether 139 subjects (68%) learned pulse palpation and performed regular pulse monitoring during the first month of follow-up, while 66 patients had inadequate capability/motivation. The demographic data of the study participants divided by the success of pulse palpation education are presented in Table 1. High MMSE Score, computer use at home, independence at daily activities and low heart rate were the independent predictors of learning pulse palpation and being motivated to continue the self-detection of heart beat at a one-month follow-up as described previously [14].

**Table 1:** Demographic data of study subjects at enrolment

N	Good motivation and capability^a^ 139	Inadequate motivation and/or capability^b^ 66	*p* value
Age, yrs	77.8 [3.3]	79.4 [5.8]	0.002
Women	78 (56.1)	38 (57.6)	0.881
BMI	26.1 [4.1]	26.6 [6.8]	0.308
Heart rate	61.6 ± 9.3	66.1 ± 9.2	0.001
Hypertension	75 (54.0)	36 (54.5)	1.000
Diabetes	20 (14.4)	15 (22.7)	0.165
Coronary artery disease	20 (14.4)	16 (24.2)	0.115
Dyslipidaemia	53 (38.1)	21 (31.8)	0.438
Previous stroke or TIA	14 (10.1)	14 (21.2)	0.048
Heart failure	4 (2.9)	2 (3.0)	1.000
Paroxysmal AF or atrial flutter	4 (2.9)	3 (4.5)	0.683
Smoking			0.946
Never smoked	109 (78.4)	53 (80.3)	
Ex-smoker	28 (20.1)	12 (18.2)	
Current smoker	2 (1.4)	1 (1.5)	
Alcohol consumption			0.970
Never	43 (30.9)	21 (31.8)	
Less than weekly	72 (51.8)	33 (50.0)	
Weekly	24 (17.3)	12 (18.2)	
MMSE Test Score	29.0 [2.0]	28.0 [4.0]	<0.001
Functional independence	134 (96.4)	53 (80.3)	<0.001
Weekly physical exercise	112 (80.6)	46 (69.7)	0.109
EQ-5D VAS	80.0 [15.0]	70.0 [30.0]	<0.001
Education level			0.007
Basic^c^	71 (51.1)	47 (71.2)	
Secondary or higher^d^	68 (48.9)	19 (28.8)	
Computer at home	63 (45.3)	10 (15.2)	<0.001

*Abbreviations*: *BMI* body mass index, *TIA* transient ischaemic attack, *MMSE* Mini-Mental State Examination

Values are presented as number (%), median [interquartile range] or mean ± standard deviation

^a^Good pulse palpation capacity (ability to find the pulse and to calculate heart beat) and follow-up diary readings completed >80%

^b^Moderate (difficulties to find pulse or calculate heart beat) or poor (inability to find pulse or calculate the heart beat) pulse palpation capacity and/or follow-up diary readings completed ≤80%

^c^≤9 years of full-time education

^d^>9 years of full-time education

Altogether 39 out of the 205 participants (19.0%) interrupted the study or did not respond to follow-up letters by the three-year follow-up. Drop-out was significantly more common in those with inadequate motivation/capability than in those with good motivation/capability (37.9% vs. 10.1%, *p* < 0.001).

### Persistence of pulse self-assessment activity

At the four months’ follow-up, 112 (80.6%) subjects with good motivation/capability and 26 (39.4%) with inadequate motivation/capability continued self-assessment of pulse daily. Although at this time 147 (71.7%) of all patients declared their commitment to continue regular pulse palpation, at 12 months only 17 (8.3%) palpated their pulse daily, 103 (50.2%) weekly and 54 (26.3%) less often but occasionally. Pulse palpation activity at 12 months did not differ significantly between the two groups (*p* = 0.075). Weekly physical exercise (OR: 2.84; 95% CI: 1.41–5.69; *p* = 0.003) and high MMSE score (OR: 1.19; 95% CI: 1.02–1.38; *p* = 0.025) were the independent predictors of continuing pulse palpation at least weekly at 12 months. At the 36 months’ follow-up, 12 (5.9%) patients palpated their pulse daily, 57 (27.8%) weekly and 70 (34.1%) less often but occasionally. Pulse palpation activity at 36 months did not differ significantly between the two groups (*p* = 0.41). There were no independent predictors for continuing pulse palpation at least weekly at 36 months.

### Self-detected pulse irregularity and outcome events

Altogether 34 (16.6%) subjects reported irregular pulse findings during the first month, 44 (21.5%) during the first four months, 55 (26.8%) during the first year and 67 (32.7%) during the three years of follow-up. New AF was found in 10 (4.9%), ischaemic stroke or TIA in 9 (4.4%) and death occurred in 11 (5.4%) subjects. A pacemaker was implanted in 4 (2.0%) subjects due to advanced atrioventricular block. New AF was diagnosed directly after observing pulse irregularity in 1 (0.5%) subject, during routine visits to the study centre in 3 (1.5%) subjects, concomitantly with an ischaemic stroke in 1 (0.5%) subject, due to palpitations in 4 (2.0%) subjects and during a routine check-up at the health centre in 1 (0.5%) subject. Oral anticoagulation therapy was initiated in all 10 of those with a new AF diagnosis. Irregular pulse findings were significantly more common in subjects diagnosed with new AF than in those who were not: 7/10 (80.0%) vs. 60/195 (30.9%) (*p* = 0.015). Conversely, new AF was diagnosed in 7 (10.4%) subjects who reported irregular pulse findings and in 3 (2.2%) subjects who did not (p = 0.015). Irregular pulse findings were not significantly associated with the composite outcome variable (*p* = 0.06), stroke/TIA (*p* = 0.48), pacemaker implantation (*p* = 0.10) or death (*p* = 1.00). The independent predictors of pulse irregularity and AF are presented in Table 2, and a list of the outcome events excepting death and bradyarrhythmia needing pacemaker implantation is provided in Additional file 1: Table S1.

**Table 2:** The independent predictors of pulse irregularity and atrial fibrillation

	Univariate *p* value	Multivariate *p* value	OR	95% CI
Pulse irregularity				
No history of diabetes	0.010	0.003	6.00	1.85–19.52
Low EQ-5D-3L	0.014	0.029	12.3	1.29–116.89
High BMI	0.077	0.034	1.09	1.01–1.19
Atrial fibrillation				
Irregular pulse findings	0.015	0.019	5.25	1.31–21.00

*Abbreviations*: *OR* odds ratio, *CI* confidence interval, *BMI* body mass index

During the stepped care subgroup intervention in 20 subjects with pulse irregularities, a total of 252 successful ECG recordings were performed during a seven day period. Ventricular extrasystoles were present in 110 recordings in 17 subjects, supraventricular extrasystoles in 142 recordings in 19 subjects and pronounced sinus arrhythmia in 69 recordings in 16 subjects. One subject had sinoatrial block in 16 recordings. No other rhythm disturbances, including atrial fibrillation and flutter, were detected.

### Quality of life

**Fig. 2:**
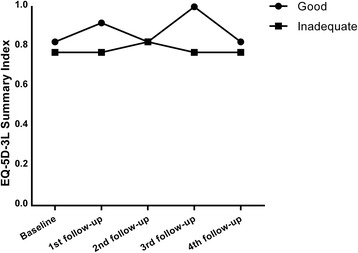
The EQ-5D-3L summary index of the subjects during the study

The EQ-5D-3L summary index is shown in Fig. 2. No significant change could be observed in the anxiety/depression dimension of the EQ-5D-3L system either in those with good (*p* = 0.26) or inadequate (*p* = 0.71) motivation/capability during the three years of follow-up. Additionally, no change was observed in the EQ-5D-3L summary index (*p* = 0.14), EQ VAS (*p* = 0.68) or the anxiety/depression dimension of the EQ-5D-3L system (*p* = 0.95) during the follow-up in those subjects who continued pulse palpation at least weekly at 36 months.

### Health care burden

No statistically significant changes were observed either in the number of visits to the municipal primary healthcare centre (*p* = 0.39) or to special healthcare outpatient clinics (*p* = 0.072) during the follow-up.

## Discussion

This study demonstrates that a simple and short nurse-led education is effective in training pulse palpation to the elderly, but also that it is challenging to maintain sufficient motivation to continue regular pulse palpation beyond a few months. Secondly, self-palpation of pulse does not affect quality of life or experienced anxiety, nor does it increase health-care burden. A total of 10 (4.9%) cases of new AF were diagnosed during the three years of follow-up, but while 7 of them had detected pulse irregularities, only 1 (0.5%) subject sought urgent medical attention and was diagnosed with AF due to self-detected irregularity. Additionally, 3 (1.5%) cases of new AF were diagnosed during visits to the study nurse.

A key manifestation of AF is pulse irregularity. The current recommendation for screening of AF is opportunistic pulse palpation by healthcare professionals in patients aged 65 years or older, followed by the recording of an ECG when irregularity is detected [13]. In 2007 Fitzmaurice et al. compared opportunistic and systematic screening with a single ECG recording to standard practice during a 12 month period in a population of 14,802 subjects aged 65 years or more [16]. The rate of new AF diagnoses in the standard care arm was 1.0%, while screening in the opportunistic and systematic arms produced similar detection rates of 1.6% [16]. Another study of 7262 subjects had a lower (0.76%) yield of new AF diagnoses detected through opportunistic screening during a shorter 6 month period, although no comparisons were made with any alternative approach [17]. According to a previous systematic review, pulse palpation has high sensitivity but low specificity for AF [18]. Consequently, pulse palpation could feasibly be used to find the subjects to whom more laborious methods, such as ECG and Holter recordings, could be targeted. The major caveat of opportunistic screening is that healthcare visits by individuals are usually rare and far apart and present only a narrow window to catch a paroxysmal arrhythmia. Regular self-assessment of pulse could potentially overcome this problem. According to Munschauer et al., members of the general public are capable of recognizing an irregular pulse with a sensitivity of 76% and specificity of 86% [19].

Although 3/4 of our subjects learned to palpate their pulse, the persistence of palpation during the intervention was disappointingly poor. After 3–4 months, 67% of the subjects palpated their pulse daily, but at 12 months, this figure was 8% and at 36 months merely 34% continued palpation at least weekly. Four months of diary keeping proved ineffective in encouraging habit formation. It may be speculated that better persistence could have been achieved by certain measures. Perhaps twice-daily palpation was too strenuous and discouraged continuing palpation in the long term or the subjects would have benefited from further follow-up visits. Previous evidence suggests that long-lasting habits, which persist even after the initial motivation to perform the behaviour has waned, are formed when a desired action is associated with an often-encountered external context, which subsequently triggers the action automatically after a short initial learning phase [20]. Until the second follow-up, the subjects had a strong motivation to continue active palpation in the form of the diary, after which continuing regular palpation depended on their own personal motivations, which inevitably fade in the long-term. Had we asked them from the beginning to associate palpating their pulse with a daily activity, better long-term results might have been achieved. Evidence suggests that habit formation is not a long process: in a study of 96 participants reporting subjective automaticity of different contextual behaviours, after an asymptotic increase, a plateau was reached after a mean of 66 days, although the interindividual variation was wide [21].

During the follow-up, pulse irregularity was reported by 1/3 of all subjects. Unfortunately, our study does not identify the events following detected irregularity and it is unclear whether the subjects appropriately contacted healthcare professionals. Although 70% of those diagnosed with AF had reported pulse irregularity, which was also an independent predictor of new AF, only one was diagnosed with AF because of it. It may either be that the subjects have not reacted appropriately or that pulse palpation followed by healthcare work-up is an ineffective screening method for one reason or another. A significant point of consideration is that, with self-detection of pulse irregularity there is an inevitable delay before an ECG recording can be performed, which allows time for an AF paroxysm to subside. Pulse irregularity due to frequent extrasystoles may also be problematic to interpret even for healthcare professionals. The significance of the intervention may also be unclear to the subjects. According to current evidence, patients with AF have limited knowledge of their condition and its implications [22, 23]. A disappointing observation was that new AF was diagnosed in conjunction with a stroke/TIA also in one patient. Nevertheless, our finding is encouraging regarding the possibility of using self-palpation of pulse to choose those who are to be screened more thoroughly for AF.

The main concerns regarding our intervention were whether it would negatively impact quality of life, cause increased anxiety or lead to unnecessary healthcare contacts. However, there was no discernible change in quality of life during the intervention, and furthermore, no change was observed in those with good adherence to pulse palpation. Moreover, no change was observed in measures of experienced anxiety. Similarly, no significant increase in the use of healthcare services was observed. It should, however, be noted that the subjects’ poor adherence to palpation may affect the reliability of these results.

Benito et al. have conducted a pilot study employing pulse self-palpation in screening of AF [24]. Subjects with risk factors of AF were divided into intervention and control groups. The intervention group were educated on symptoms of AF, taught to palpate their pulse monthly and seek medical attention as needed. Additionally, office visits including medical history taking, physical examination and recording an ECG occurred every six months. After two years, 2.5% of the subjects in the intervention and 1.3% in the control group were diagnosed with new AF. Although these results are promising, in the light of our findings, pulse palpation probably was not a major factor in achieving better yield in the intervention group.

Utilizing portable ECG recorders after self-detected pulse irregularity might be an effective and cost-efficient screening model provided that better persistence of pulse palpation and adequate reporting could be promoted. Accordingly, we performed a supplementary intervention with portable ECG recorders in patients with self-detected pulse irregularities, but disappointingly no new cases of AF were detected. The cohort size was, however, only 20 subjects and the length of intervention one week. Lowres et al. conducted a study where 1000 subjects aged at least 65 were screened at a single time point for AF with an iPhone-based ECG recorder by pharmacists. Altogether 15 (1.5%) cases of new AF were detected, which is comparable to the rate achieved by opportunistic screening [25]. Another screening study utilizing iPhone-based hand-held ECG recorders was conducted by the same research group on 42 subjects who had experienced paroxysmal AF after cardiothoracic surgery but had no previous history of AF and were discharged in stable SR. The results were encouraging: it was demonstrated that, after education, the independent use of hand-held iPhone-based ECG recorders was feasible in the screening of AF and well-accepted by the target population during a four-week follow-up, while recurrence of AF was detected in as many as 24% of the subjects [26]. The greatest yield of new AF has been achieved by the STROKESTOP investigators using portable ECG recorders in systematic screening of AF. In all, 7173 subjects belonging to the general 75–76 year-old population were screened for AF with a standard ECG recording followed by two weeks of ECG recordings performed twice-daily and when palpitations are noticed [27]. New AF was found in 3.0% of the subjects, after which the prevalence of AF in the study population was 12.3%. In only 0.5% of the screened population new AF was detected on the initial ECG. Single time-point screening seems inefficient and repeated ECG recordings are needed for better results. Previous evidence suggests that brief intermittent ECG recordings have a better detection rate of AF compared to Holter recordings [28].

Some limitations still need to be addressed. A control group receiving usual care was not included in our study, which is a notable limitation. This approach was chosen mainly due to the small population base of Lieto, which would probably have led to any control group being contaminated by the information relayed to the intervention group. Our study was also limited by 19% of subjects dropping out or not responding to follow-up letters by 36 months of follow-up.

## Conclusion

Although pulse palpation is a skill that can be learned even at an older age, it is challenging to maintain a continuing habit of monitoring heart beat on a daily basis. Consequently, self-monitoring of pulse regularity is of limited value in the screening of AF unless better persistence of active palpation can be promoted and an efficient and clearly defined follow-up algorithm to detected irregularity is devised. With these preconditions pulse palpation might be an adequate preliminary screening method before more laborious and expensive methods are used. Research to explore these possibilities and alternative screening methods and strategies are needed.

## Additional file

Additional file 1: Table S1. List of the study outcome events excepting death and bradyarrhythmia requiring pacemaker implantation. (DOCX 16 kb)

## Abbreviations

AF: Atrial fibrillation
BMI: Body mass index
CI: Confidence interval
MMSE: Mini-Mental State Examination
OR: Odds ratio
TIA: Transient ischemic attack
VAS: Visual analogue scale

## References

[1] Wolf PA, Abbott RD, Kannel WB (1991). Atrial fibrillation as an independent risk factor for stroke: the Framingham study. Stroke.

[2] Wolf PA, Abbott RD, Kannel WB (1987). Atrial fibrillation: a major contributor to stroke in the elderly: the Framingham study. Arch Intern Med.

[3] Marini C, De Santis F, Sacco S, Russo T, Olivieri L, Totaro R (2005). Contribution of atrial fibrillation to incidence and outcome of ischemic stroke results from a population-based study. Stroke.

[4] Kolominsky-Rabas PL, Weber M, Gefeller O, Neundoerfer B, Heuschmann PU (2001). Epidemiology of ischemic stroke subtypes according to TOAST criteria incidence, recurrence, and long-term survival in ischemic stroke subtypes: a population-based study. Stroke.

[5] Sanna T, Diener HC, Passman RS, Di Lazzaro V, Bernstein RA, Morillo CA (2014). Cryptogenic stroke and underlying atrial fibrillation. N Engl J Med.

[6] Gladstone DJ, Spring M, Dorian P, Panzov V, Thorpe KE, Math M (2014). Atrial fibrillation in patients with cryptogenic stroke. N Engl J Med.

[7] Go AS, Hylek EM, Phillips KA, Chang Y, Henault LE, Selby JV, Singer DE (2011). Prevalence of diagnosed atrial fibrillation in adults: national implications for rhythm management and stroke prevention: the AnTicoagulation and risk factors in atrial fibrillation (ATRIA) study. JAMA.

[8] Miyasaka Y, Barnes ME, Gersh BJ, Cha SS, Bailey KR, Abhayaratna WP (2006). Secular trends in incidence of atrial fibrillation in Olmsted County, Minnesota, 1980 to 2000, and implications on the projections for future prevalence. Circulation.

[9] Yiin GS, Howard DP, Paul NL, Li L, Luengo-Fernandez R, Bull LM (2014). Age-specific incidence, outcome, cost, and projected future burden of atrial fibrillation-related embolic vascular events: a population-based study. Circulation.

[10] Savelieva I, Camm AJ (2000). Clinical relevance of silent atrial fibrillation: prevalence, prognosis, quality of life, and management. J Interv Card Electrophysiol.

[11] Jaakkola J, Mustonen P, Kivinemi T, Hartikainen JEK, Palomäki A, Hartikainen P, et al. Stroke as the first manifestation of atrial fibrillation. PLoS One. 2016; doi:10.1371/journal.pone.0168010. 10.1371/journal.pone.0168010 PMC5148080 27936187

[12] Hart RG, Benavente O, McBride R, Pearce LA (1999). Antithrombotic therapy to prevent stroke in patients with atrial fibrillation: a meta-analysis. Ann Intern Med.

[13] Camm AJ, Lip GY, De Caterina R, Savelieva I, Atar D, Hohnloser SH (2012). 2012 Focused update of the ESC guidelines for the management of atrial fibrillation: an update of the 2010 ESC guidelines for the management of atrial fibrillation. Developed with the special contribution of the European heart rhythm association. Europace.

[14] Virtanen R, Kryssi V, Vasankari T, Salminen M, Kivelä SL, Airaksinen KEJ (2014). Self-detection of atrial fibrillation in an aged population: the LietoAF study. Eur J Prev Cardiol.

[15] Rabin R, Charro FD (2001). EQ-SD: a measure of health status from the EuroQol group. Ann Med.

[16] Fitzmaurice DA, Hobbs FR, Jowett S, Mant J, Murray ET, Holder R (2007). Screening versus routine practice in detection of atrial fibrillation in patients aged 65 or over: cluster randomised controlled trial. BMJ.

[17] Smyth B, Marsden P, Corcoran R, Walsh R, Brennan C, McSharry K, et al. Opportunistic screening for atrial fibrillation in a rural area. QJM. Epub ahead of print 27 January 2016. doi:10.1093/qjmed/hcw011. 10.1093/qjmed/hcw011 PMC4986429 26819299

[18] Cooke G, Doust J, Sanders S (2006). Is pulse palpation helpful in detecting atrial fibrillation? A systematic review: particular high-risk patients may benefit from repeated testing. J Fam Pract.

[19] Munschauer FE, Hens MM, Priore RL, Stolarski E, Buffamonte S, Carlin A (1999). Screening for atrial fibrillation in the community: a multicenter validation trial. J Stroke Cerebrovasc Dis.

[20] Gardner B, Lally P, Wardle J (2012). Making health habitual: the psychology of ‘habit-formation’ and general practice. Br J Gen Pract.

[21] Lally P, Van Jaarsveld CH, Potts HW, Wardle J (2010). How are habits formed: modelling habit formation in the real world. Eur J Soc Psychol.

[22] McCabe PJ, Schad S, Hampton A, Holland DE (2008). Knowledge and self-management behaviors of patients with recently detected atrial fibrillation. Heart Lung.

[23] Lane DA, Ponsford J, Shelley A, Sirpal A, Lip GYH (2006). Patient knowledge and perceptions of atrial fibrillation and anticoagulant therapy: effects of an educational intervention programme: the West Birmingham atrial fibrillation project. Int J Cardiol.

[24] Benito L, Coll-Vinent B, Gómez E, Martí D, Mitjavila J, Torres F (2015). EARLY: a pilot study on early diagnosis of atrial fibrillation in a primary healthcare centre. Europace.

[25] Lowres N, Neubeck L, Salkeld G, Krass I, McLachlan AJ, Redfern J (2014). Feasibility and cost-effectiveness of stroke prevention through community screening for atrial fibrillation using iPhone ECG in pharmacies. The SEARCH-AF study Thromb Haemost.

[26] Lowres N, Mulcahy G, Gallagher R, Freedman SB, Marshman D, Kirkness A (2016). Self-monitoring for atrial fibrillation recurrence in the discharge period post-cardiac surgery using an iPhone electrocardiogram. Eur J Cardiothorac Surg.

[27] Svennberg E, Engdahl J, Al-Khalili F, Friberg L, Frykman V, Rosenqvist M (2015). Mass screening for untreated atrial fibrillation: the STROKESTOP study. Circulation.

[28] Sobocinski PD, Rooth EÄ, Kull VF, von Arbin M, Wallén H, Rosenqvist M (2012). Improved screening for silent atrial fibrillation after ischaemic stroke. Europace.

