# Supplementary material for: Self-detection of atrial fibrillation in an aged population: three-year follow-up of the LietoAF intervention study
Source: BMC Geriatr. 2017 Sep 16;17:218. doi: 10.1186/s12877-017-0607-0 (PMC5602855; doi:10.1186/s12877-017-0607-0)
Supplement: Additional file 1: Table S1. — List of the study outcome events excepting death and bradyarrhythmia requiring pacemaker implantation. (DOCX 16 kb) [file 12877_2017_607_MOESM1_ESM.docx]

Table S1. List of the study outcome events excepting death and bradyarrhythmia requiring pacemaker implantation.

| Outcome event | Patient | Timing (days) | Event description |
| --- | --- | --- | --- |
| Atrial fibrillation | Male, 77 | 0 | AF was detected during the baseline visit. |
|  | Male, 76 | 0 | AF was detected during the baseline visit. |
|  | Female, 77 | 28 | AF was detected during the one month’s follow-up visit. |
|  | Male, 86 | 53 | AF was diagnosed concomitantly with an embolic ischemic stroke of the territory supplied by the left middle cerebral artery. |
|  | Female, 77 | 385 | AF was diagnosed at the emergency room after the patient had experienced palpitations daily for several months. |
|  | Female, 84 | 483 | AF was diagnosed at the emergency room after the sudden onset of palpitations. |
|  | Female, 77 | 550 | AF was diagnosed at the emergency room after the patient had noticed pulse irregularity. The patient had suffered from tiredness and exercise intolerance for several months previously. |
|  | Male, 83 | 952 | AF was diagnosed during a routine check-up for hypertension at a municipal health centre. |
|  | Female, 82 | 996 | AF was diagnosed at the emergency room after the patient had noted fluctuating heart rates and blood pressure readings for some time. |
|  | Male, 79 | 1028 | AF was diagnosed at a municipal health centre after the patient had experienced a fast and irregular heart beat for more than a day. |
| Ischaemic stroke | Male, 86 | 53 | The patient suffered an embolic ischaemic stroke of the territory supplied by the left MCA, which presented with right-sided hemiparesis. AF was diagnosed concomitantly. |
|  | Female, 77 | 368 | Occlusion of the right MCA stem was detected after the patient presented with dizziness, left-sided hemiparesis and dysarthria among other symptoms. |
|  | Female, 85 | 673 | The patient suffered an ischaemic stroke of the territory supplied by the left MCA, which presented with right-sided hemiparesis and aphasia. The patient died at 773 days due to complications of the stroke. |
|  | Male, 76 | 744 | After presenting with right visual field loss of vision, left-sided occipital ischaemic stroke was diagnosed. |
|  | Female, 76 | 751 | The patient presented with right sided hemiparesis and facial paresis as well as dysarthria. After the head CT scan proved negative, the patient received thrombolysis treatment. Subsequent CT scans remained negative and the event was considered clinically a lacunar stroke by the attending neurologist. |
|  | Male, 81 | 831 | After the patient presented with right-sided hemiparesis, multiple infarctions over several vascular territories were detected in an MRI scan. |
|  | Male, 76 | 865 | The patient experienced a sudden onset of fluctuating double vision and saccadic eye movements. The head CT scan examined acutely was negative, but the event was considered by the attending neurologist clinically an ischaemic stroke of the brainstem. |
| TIA | Female, 75 | 282 | The patient presented with hemiparesis of the left hand lasting 15 minutes and was diagnosed with carotid region TIA. |
|  | Female, 90 | 756 | The patient presented with aphasia and hemiplegia lasting 30 minutes and was diagnosed with carotid region TIA. The patient had a history of paroxysmal AF. |

Abbreviations: AF, atrial fibrillation; MCA, middle cerebral artery; CT, computed tomography; MRI, magnetic resonance imaging; TIA, transient ischaemic attack
